# The Incubation of Plague

**Published:** 1900-06-09

**Authors:** 


### THE INCUBATION OF PLAGUE.

From a careful consideration of many facts collected from various sources, Dr. Frank Clemow[1] says that it may be asserted that when plague is contracted through a wound or abrasion received during a necropsy on a plague cadaver, or through the conjunctiva, the incubation period is usually two to three days, and is probably somewhat shorter than when contracted in the more usual way. When contracted in the ordinary manner the interval between exposure to infection and the onset of symptoms is usually from two to seven days, but it may be as short as 24 hours; and it may be extended in rare cases to a period of several weeks, in which cases it is reasonable to believe that the virus has for a considerable portion of the intervening time been preserved in fomites outside the body of the patient. For practical purposes it has been generally agreed to regard the maximum incubation period of plague as 10 or 12 days.

[1] Lancet, May 26.

